# Investigation of relationship between quality of working life and organizational commitment of nurses in teaching hospitals in Tabriz in 2014

**Published:** 2015

**Authors:** D Ghoddoosi-Nejad, E Baghban Baghestan, A Janati, A Imani, Z Mansoorizadeh

**Affiliations:** * Social Determinants of Health Research Center, Birjand University of Medical Sciences, Birjand, Iran; ** Social Determinants of Health Research Center, Birjand University of Medical Sciences, Birjand, Iran; *** Department of Health Services Management, Iranian Center of Excellence in Health Management, School of Management and Medical Informatics, Student Research Committee, Tabriz University of Medical Sciences, Tabriz, Iran

**Keywords:** quality of life, organizational commitment, nurses, health training

## Abstract

The current research aimed to investigate the link between the quality of working life and the systematic commitment of nurses in the teaching hospitals in Tabriz. The methodology used was functional regarding the purpose and the proportional allocation as far as the stratified sampling method was concerned. The study population consisted of all the nurses in Tabriz. The instrument used in this study was a standard questionnaire, whose reliability was approved in national and international studies. Also data were collected and inserted into SPSS 20 software and a statistical analysis was performed. The results showed that the individuals’ quality of working life had a direct effect on their action in the organization.

## Introduction

One of the factors affecting the performance of human resources in the hospital is having systematic commitment. Systematic commitment was described in various methods such as other systematic behavior. Porter and colleagues (1974) [**1**] defined the organizational commitment as accepting the organization’s values and engaging in the organization, and knowing that its measurement criteria include motivation, desire to continue working and acceptance of the values of the organization. Allen and Meyer (1977) [**4**,**10**] defined organizational commitment as a kind of mental state which indicated the desire and need or requirement to continue serving in an organization. From their perspective, the organizational commitment includes emotional commitment, continued commitment, and normative commitment [**2**]. The most common way of dealing with the systematic commitment is that, the organizational commitment is considered a feeling attachment to a systematic, or stated as a loyalty sense to the system [**1**]. Based on the theories, a committed person (who had the least emotional commitment) did not merely have a physical presence in the organization, but tried, in the interest of the organization [**3**]. The presence of committed manpower showed that the image of the organization is important in the society, providing the background for the organization’s growth and development [**4**]. The direct effect of systematic commitment on the systematic action has been confirmed in many studies. There are many people who are less committed going out and being absent from work [**1**]. The quality of working life programs involves any enhancement in the systematic behavior that promotes the excellence and growth of the employees in the organization [**5**]. One of the most important issues in human resource management in organizations is providing needs and motivating people to enhance the quality of their work [**6**]. On the other hand, meeting the needs of staff leads to an improvement and long-term efficiency [**5**]. The organization that attends the quality of working life of its employees will benefit from having a committed workforce and the commitment of the workforce means a higher productivity of workforce [**2**].

The quality of working life is a series of actions and revisions that provide their long-term interests of individuals, organizations and society scholar through improved working conditions, job enrichment, employee empowerment and enhancement of their knowledge, insight and professional skills [**3**,**7**].

**Theoretical Foundations and research background**

**Theoretical Foundations**

**Definition of quality of working life **

The quality of working life represents the reactions of the employees towards work, in particular individual consequences of the job satisfaction and mental health. The quality of working life is the employees’ ability to satisfy important personal needs by using the experiences gained in that organization, this definition strictly emphasizing on creating an environment which leads to the satisfaction of the needs of individuals.

**Components of Walton quality of working life**

Walton has set a model to explain the quality of working life: 

1. Good and adequate payment: identical payment for identical job and also suitability of payments in terms of social norms and standards of workers as well as its suitability for other types of work;

2. Healthy and secure conditions: the creation of safe physically working conditions and the determination of reasonable working hours; 

3. Supplying chances for continuous security and growth: supplying background for the improvement of individual ability, opportunities for advancement, chances to employ the needed experiments and secure income and employment;

4. Legality in the organization: to provide freedom of speech without any problem of an official reaction and domination of penetrating the rule of law through man;

5. The social dependence of working life: perception (understanding) way of employees;

6. The general atmosphere of life: generation of balance between the working life and the rest of life parts including education, leisure, and family;

7. Integration and social cohesion in the organization of work: creating the right working atmosphere, that strengthens the sense of relating to the system’s workspace and that, they are required by the organizations.

8. Development of human capabilities: the availability of chances like self-control and independence within the job, advantage of the diverse experiments and receiving information related with job [**8**].

**Types of organizational commitment**

Allen and Meyer (1997) believed that the commitment links the individual to the organization and this link will reduce the probability of turnover (Meyer and Herskovic, 2002). They have provided three components for the organizational commitment:

1. Emotional commitment: including an emotional link between the employees and the organization, so that people could introduce themselves to their organization.

2. The continuous commitment: according to this commitment, an individual calculates the cost of leaving the organization. In fact, one wonders what costs will be incurred if you leave the organization. In fact, people who are committed in a continuous form are people who stay in the organization because they need to stay.

3. Normative commitment: In this form, an employee feels that he should stay and this decision is the right action [**9**].

**Research background**

In a study entitled “The analysis of the link among systematic commitment, job satisfaction, and happiness”, Kamalizadeh, Khosravi and Soghad (2012) investigated the relationship between organizational commitment, quality of working life and happiness in the nursing staff of Namazi Hospital in Shiraz. The research method was descriptive correlational, and the community included all the nurses of Namazi Hospital, 200 of these being chosen with the available sampling method. Data collection was conducted by using Stiriz and Porter (1979) material organizational commitment questionnaire, Ghasemzadeh (2005) component of separation of the quality of working life and Oxford Martin et al. (1987) happiness concept. According to the research conducted by Hamidi (2003), Winhoven (1994) and Rezaei (2000), it was shown that the employee satisfaction leads to happiness and an increase in the quality of working life, and it could have an effect on the organizational commitment. According to the discussions, three hypotheses and a research question were developed, each of them being analyzed [**10**].

**Research hypothesis**

1- The main theory: there was a link between the quality of working life and the commitment of systematic of nurses of Tabriz teaching hospitals.

2- Subsidiary hypothesis 1: there was a link among QWL (1- fair pay 2- legalism 3- supplying chances for sustainable safety and growth 4- social dependence 5- development of the individual abilities 6- general workspace 7- environmental safety 8- and social integration) and the organizational commitment of nurses in teaching hospitals in Tabriz.

3- Subsidiary hypothesis 2: There was a difference in the mean score of systematic commitment and its sizes regarding the demographic variables of gender group, marital status, age, work experience group, and working in a hospital of nurses in the teaching hospitals in Tabriz.

**Research methodology**

This is a descriptive study performed in a cross-sectional form and, given the nature of the research, the stratified sampling method was used in the form of proportional allocation. The instruments used in this research were two-page questionnaires, whose reliability and validity has been proven already. Finally, the data were collected and inserted into SPSS 20 software and were analyzed by using this software.

**Findings and Interpretation of Results**

***a. Evaluation of descriptive information of people participating in the study***

The table below shows that the average age of nurses was of approximately 35 and the amount of work experience was 11.

**Table 1:** Table of descriptive statistics for the variables of age and work experience of nurses participating in the study

Variable	Average	SD	Min	Max
age	35.12	6.882	24	53
Work experience	11.10	6.497	1	29

The results in **Table 2** show that the participation of most nurses in the study was from Imam Reza Hospital, the lowest being associated with Taleghani and the sample selection was made based on the proportional allocation to population size.

**Table 2:** Distribution of nurses involved in the study separated according to the hospital

Hospital	Frequency	Relative Frequency percent
Emam Reza	86	36.4
Shahid Madani	72	30.5
Sina	40	16.9
Children’s Hospital	27	11.4
Taleghani	11	4.7
Total	236	100.0

Tables and Figures of the following age ranges show that most people were in the age group of 32 to 40 and the minimum age was 40 years.

**Table 3:** Age range of nurses who contributed to the study

Age range	Frequency	Relative frequency percent	Cumulative frequency percent
24-32	79	33.9	33.9
32-40	93	33.9	73.8
40-48	47	20.2	94.0
48-56	14	6.0	100.0
Total	233	100.0	

The results showed that most of the nurses were women.

**Table 4:** Gender composition of the nurses who took part in the study

Gender group	Frequency	Relative frequency percent
Female	209	88.6
Male	27	11.4
Total	236	100.0

Approximately 50% of the nurses had between 5 and 15 work experience and the remaining 45 percent was distributed in a high record, recording low groups almost equally.

**Table 5:** Distribution of nurses participating in the study based on work experience

Work experience	Frequency	Relative frequency percent	Cumulative frequency percent
1-5	51	22.5	22.5
5-15	127	55.9	78.4
15-25	45	19.8	98.2
=>25	4	1.8	100.0
Total	227	100.0	

The following results show that most participants in the study were married.

**Table 6:** Distribution of nurses taking part in the study based on marital status

Marital status	Frequency	Relative frequency percent
Single	51	21.6
Married	181	76.7
Other	4	1.7
Total	236	100.0

The majority of nurses had a bachelor’s degree and only 8% had higher degrees.

**Table 7:** Distribution of nurses taking part in the study based on education status

Education status	Frequency	Relative frequency percent
Single	217	91.9
Married	19	8.1
Total	236	100.0

**Descriptive indicators of the quality of life and the organizational commitment variables**

The results showed that the average quality of life of nurses was 32% and, according to their comments, their level of organizational commitment was 42%. In any case, the individual commitment was slightly higher than the quality of life, which may represent individual responsibility. Other indicators also showed that the values of the two variables did not have further changes and fluctuations and the approximate values were around their average value.

**Table 8:** Descriptive statistics for the variables of quality of life and organizational commitment

Statistics	Quality of life	Organizational commitment
Mean	32.0851	42.1697
95% SD	(30.5641, 33.6061)	(40.5474, 43.7919)
5% trimmed mean	32.2521	42.9292
Middle	33.8988	44.8306
Variance	140.668	160.018
SD	11.86034	12.64981
Minimum	3.27	1.69
Maximum	59.26	63.19
Domain	55.99	61.51
Interquartile range	16.29	15.98
Skewness	-.265	-.915
Strain	-.472	.539

**Evaluation normality of variables of quality of life and organizational commitment**

**Fig. 1:**
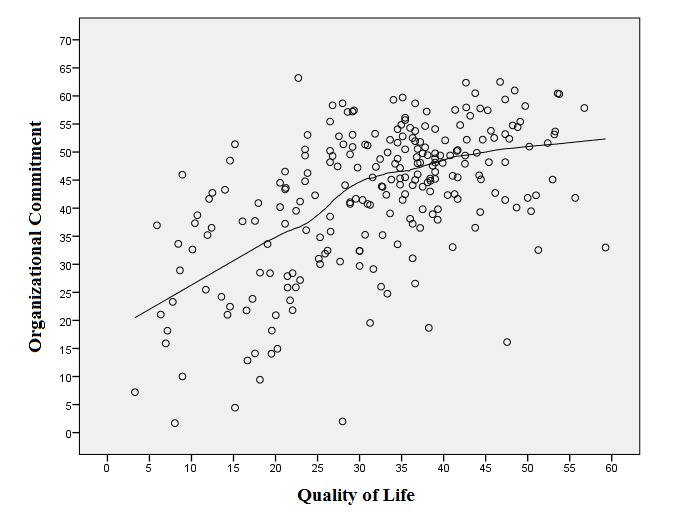
Scattering plot in Louis curve between the quality of life and the systematic commitment

**Fig. 2:**
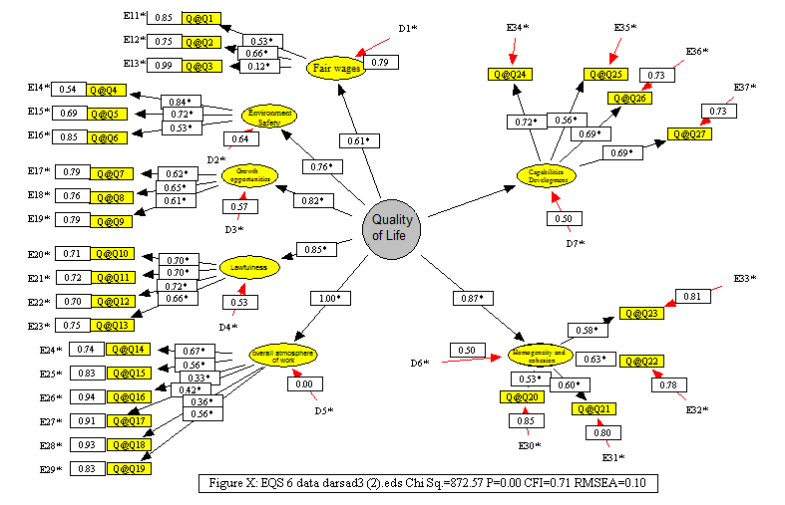
Second order factor analysis diagram of the quality of life variable in EQS software output

**Fig. 3:**
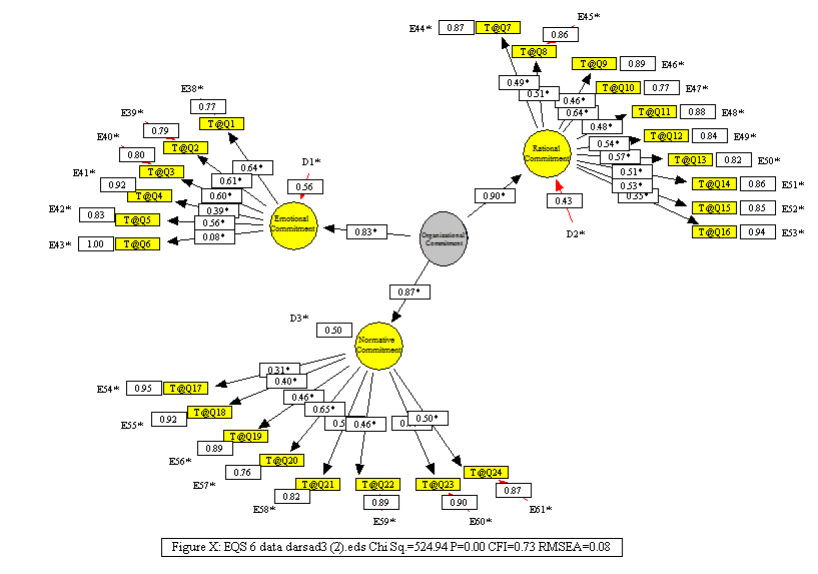
Second order factor analysis diagram of the organizational commitment variable in EQS software output

**Fig. 4:**
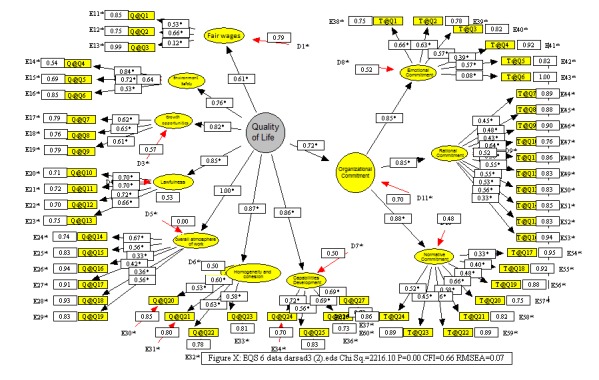
Structure model diagram of the link between the quality of life and the systematic commitment in EQS software output

**Fig. 5:**
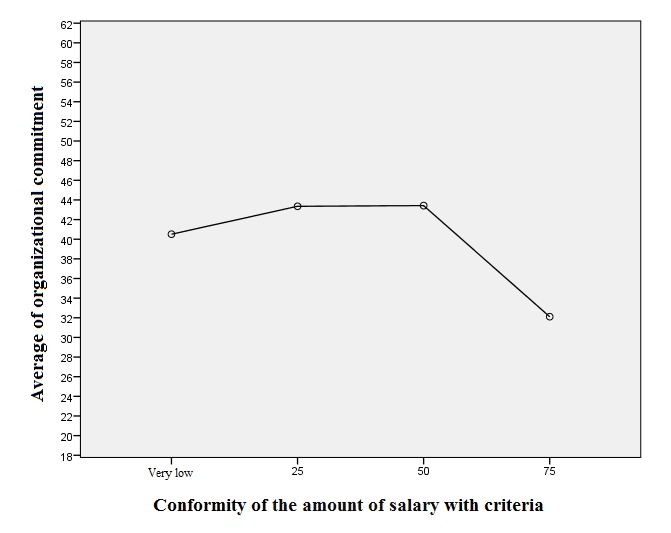
Diagram of the average trend of organizational commitment of nurses in the workplace based on the salary

**Table 9** shows Kolmogorov-Smirnov and Shapiro-Wilk test statistics to determine the normality of the data. The results showed that the variables of the quality of life and the organizational commitment have deviations from the normal distribution. This deviation from the normal state did not visually seem very clear in **Fig. 1**-**5**.

**Table 9:** Statistics of normality evaluation for the variables of quality of life and organizational commitment

Variable	Kolmogorov-Smirnov			Shapiro-Wilk		
	Statistics	DOF	P- Amount	Statistics	DOF	P- Amount
Quality of life	0.073	236	0.004	0.985	236	0.014
Organizational commitment	0.100	236	0.000	0.941	236	0.000

**Analysis of the link between the quality of life and the systematic commitment of nurses in the workplace**

**Fig. 1** shows the scattering plot of the quality of working life of nurses regarding the organizational commitment. As Louis curve and value showed, there was an upward trend in the organizational commitment with an improvement of the quality of life. Regarding this, it was necessary that the slope of the upward trend of the organizational commitment were sharp towards the quality of life, of 30%, being relatively adjusted at higher values, this indicating that the lower quality of life had an important role in increasing the organizational commitment. 

**Table 10** shows Pearson and Spearman’s correlation coefficient in confirming a clear direct link between the quality of life and the systematic commitment.

**Table 10:** Link between the quality of life and the systematic commitment

Correlation coefficient	Statistics	P-value
Pearson	.553**	<o.ooo1
Spearman	.553**	<o.ooo1

**Linear model of the effect of the quality of life on the systematic commitment of nurses at workplace**

In order to study the effect of the quality of life on an organizational commitment, a simple linear regression models was fitted to the data. The results showed the effectiveness of the quality of life on the organizational commitment.

**Table 11:** ANOVA linear regression model of the impact of the quality of life on the organizational commitment of nurses

Source of change	Sum of squares	Degrees of freedom	Mean Squares	P-value
Regression	11492.91	1	11492.912	<o.ooo1
Remaining	26111.260	234	111.587	
Total	37604.172	235		

The results of **Table 12** indicated that on average, by increasing each percent on the quality of life of nurses, a 0.59 percent is added to the organizational commitment.

**Table 12:** Significance of linear regression slope and interception of the impact of the quality of life on the organizational commitment of nurses

	Unstandardized coefficient		Standardized coefficient	T-student statistics	P-value
	Standard error of beta	Beta	Beta		
Intercept	1.987	23.251		11.702	<o.ooo1
Slope	.058	.590	.553	10.149	<o.ooo1

**Investigation of the relation between different dimensions of quality of life**

To evaluate the quality of life, a second-order factor analysis relational model was provided and it was shown in **Fig. 2**. The most important evaluation index was CFI, whose value was a bit higher than 0.95 of an ideal model. The value obtained from fitting the model on the data of this study was of approximately 0.71. Although it did not show an ideal model, the above value was considered a good value for the assessment of the model. Basically, the values of less than 0.5 indicated a worthless model. These results showed that with little or no modification in the model structure a valuable model could be achieved. This required an exploratory factor analysis that had to be performed in another study. In this model, in addition to the assessment of the whole model, the role of each variable in determining the quality of life variables could be examined. The results on the output of EQS6 software indicated a significant positive effect of each variable in determining the variable of the quality of life, which, due to the high volume, results have not been listed here. However, given that the software showed impact coefficients on the graph and indicated their significance by putting an asterisk on the coefficient, the effects of the graph could be evaluated.

**Investigation of the different dimensions of organizational commitment**

**Fig. 3** shows the significance of measuring the organizational commitment and its dimensions of all the questions. Also, the effect of each variable and its significance are shown in **Fig. 3**.

**Investigating the structural model of the link between the quality of life and the systematic commitment**

Finally, a structural equation model was used to evaluate the structural link between the quality of life and the systematic commitment with their dimensions. The general model showed a moderate relationship. The effect of each question on various aspects of the quality of life and the systematic commitment was also shown in **Fig. 4**. All the coefficients had a positive significance. As it could be seen, the effect of the quality of life on the organizational commitment was significant and the standard effect coefficient was equal to 0.72.

**Comparison of organizational commitment based on demographic information**

**Comparison of organizational commitment based on gender group**

**Table 13** , descriptive statistics, shows the level of the organizational commitment in both genders.

**Table 13:** Descriptive statistics for the organizational commitment based on gender group

Gender	Number	Mean benefit score (%)	SD of benefits (%)	Mean standard error
Female	209	41.6536	12.80162	.88551
Male	27	46.1648	10.79568	2.07763

**Table 14** shows that the organizational commitment in both genders is not significant.

**Table 14:** T-test values of two independent samples - comparison for the organizational commitment based on genders

Comparison	T statistics	Degrees of freedom	P-value	Mean difference	95% confidence interval for the difference between the mean	
					Upper limit	Lower limit
Two gender group	-1.751	234	.081	-4.51117	.56321	-9.58555

Similarly, organizational commitment of the two groups of single and married were evaluated and the results showed no significant difference.

**Comparison of organizational commitment for each hospital**

The results of **Table 15** showed that the organizational commitment of nurses in each hospital had a significant difference (P-value <o.ooo1).

**Table 15:** Variance analysis to assess the organizational commitment of nurses in each hospital, at workplace

Source of change	Sum of squares	Degrees of freedom	Mean Squares	Fischer statistic	P-value
Regression	3456.169	4	864.042	5.845	<o.ooo1
Remaining	34148.003	231	147.827		
Total	37604.172	235			

The follow-up tests also showed that the organizational commitment of nurses in Imam Reza Hospital was different from Madani (P-value <o.ooo1) and Sina (P-value = .006).

**Comparison of organizational commitment according to age group**

**Table 16** and **Fig. 5** showed that the organizational commitment was not different in different age groups.

**Table 16:** Variance analysis to assess the organizational commitment of nurses in each age group

Source of change	Sum of squares	Degrees of freedom	Mean Squares	Fischer statistic	P-value
Regression	1175.632	3	391.877	2.468	.063
Remaining	36359.334	229	158.774		
Total	37534.966	232			

**Comparison of organizational commitment based on the working experience group**

The results also showed that the level of the organizational commitment in different working experience groups did not differ significantly (P-value = 0.084).

**Table 17:** ANOVA for each working experience group to assess the organizational commitment of nurses

Source of change	Sum of squares	Degrees of freedom	Mean Squares	Fischer statistic	P-value
Regression	1061.300	3	353.767	2.246	.084
Remaining	35119.116	223	157.485		
Total	36180.416	226			

**Table 18:** ANOVA to assess the level of organizational commitment based on the salary

Source of change	Sum of squares	Degrees of freedom	Mean Squares	Fischer statistic	P-value
Regression	664.189	3	221.396	1.382	.249
Remaining	36850.817	230	160.221		
Total	37515.005	233			

**Interpretation of results**

The results showed that the quality of working life had a direct effect on their action in the organization. Therefore, the lack of attention to the quality of working life decreases the employee’s morale and increases absenteeism, turnover and psychological stress. Also, providing a quality of life of vulnerable people further guarantees an increase in the organizational commitment of this group of people at their workplace. Its importance shows the need to investigate the correlation between the nurses’ quality of working life and the systematic commitment.

## References

[1] Yaghoubi M, Yarmohammadian  MH, Javadi  M (2007). The relationship between organizational commitment and job stress among managers of teaching hospitals of Isfahan University of Medical Sciences. Scientific and Research Quarterly Journal of Health Administration.

[2] Mehrad A, Mahdavi RN, Golparvar M (2011). The relationship between quality of work life and organizational commitment and its components.

[3] Bagheri M, Tavalaee R (2010). Investigating the effect of organizational commitment on organizational performance. Bimonthly of Human Development.

[4] Delgoshaei B, Tofighi S, Kermani B (2008). The relationship between organizational atmosphere and organizational commitment of employees and managers of teaching hospitals of Hamedan University of Medical Sciences. Journal of Gonabad University of Medical Sciences and Health Services.

[5] Saedi S, Khalatbari J, Mouri Najaf AN (2010). The relationship between quality of work life and organizational health with job satisfaction. Quarterly Journal of Industrial/Organizational Psychology News.

[6] Kouzechian H, Zarei J, Talebpour (2003). Investigating the relationship between organizational commitment and job satisfaction of managers and male teachers of physical education. Olympic Journal.

[7] Hatami H, Mir Jafari SA, Mojahedi JS (2011). The relationship between quality of work life with organizational commitment and productivity amount in employees of Jahrom University of Medical Sciences. Quarterly Journal of New Approaches in Educational Administration of Islamic Azad University of Marvdasht Branch.

[8] Mirkamali SM, Narenji SF (2008). Investigating the relationship between quality of work life and job satisfaction among faculty members of Tehran University and Sharif University of Technology. Quarterly Journal of Research and Planning in Higher Education.

[9] Behravan H, Saeedi  R (2009). Factors affecting the organizational commitment amount of Gas Company Headquarters employees of Mashhad city. Journal of Social Sciences Faculty of Literature and Humanities of Mashhad Ferdowsi University.

[10] Soltan HM, Masoud  N, Reza  H, Zohreh M (2009). The relationship between quality of work life and organizational commitment of employees of Physical Education General Department of Isfahan Province. Sport Manageme.

